# Dietary Probiotic *Bacillus subtilis* AAHM-BS2360 and Its Postbiotic Metabolites Enhance Growth, Immunity, and Resistance to Edwardsiellosis in *Pangasianodon hypophthalmus*

**DOI:** 10.3390/antiox14060629

**Published:** 2025-05-23

**Authors:** Nugroho Wiratama, Pakapon Meachasompop, Benchawan Kumwan, Yosapon Adisornprasert, Prapansak Srisapoome, Phornphan Phrompanya, Patcharapong Thangsunan, Pattanapong Thangsunan, Kanokporn Saenphet, Supap Saenphet, Wararut Buncharoen, Anurak Uchuwittayakul

**Affiliations:** 1 Laboratory of Aquatic Animal Health Management, Department of Aquaculture, Faculty of Fisheries, Kasetsart University, Bangkok 10900, Thailand; nugroho.w@ku.th (N.W.); pakapon.meac@ku.th (P.M.); benchawan.kumw@ku.th (B.K.); yosapon.ad@ku.th (Y.A.); ffispssp@ku.ac.th (P.S.); 2 Center of Excellence in Aquatic Animal Health Management, Faculty of Fisheries, Kasetsart University, Bangkok 10900, Thailand; 3 PT. Central Proteinaprima, Tbk, Treasury Tower Lt. 8, District 8 SCBD Lot. 28, Senayan, Jakarta 12190, Indonesia; 4 Department of Biological Science, Faculty of Science, Ubon Ratchathani University, Warin Chamrap District, Ubon Ratchathani 34190, Thailand; phornphan_ppy@hotmail.com; 5 Office of Research Administration, Chiang Mai University, Chiang Mai 50200, Thailand; tbscience@gmail.com; 6 Division of Biochemistry and Biochemical Innovation, Department of Chemistry, Faculty of Science, Chiang Mai University, Chiang Mai 50200, Thailand; pattanapong.t@cmu.ac.th; 7 Center of Excellence for Innovation in Chemistry, and Research Laboratory on Advanced Materials for Sensor and Biosensor Innovation, Material Science Research Center, Faculty of Science, Chiang Mai University, Chiang Mai 50200, Thailand; 8 Department of Biology, Faculty of Science, Chiang Mai University, Chiang Mai 50200, Thailand; kanokporn.saenphet@cmu.ac.th (K.S.); supap.saenphet@cmu.ac.th (S.S.)

**Keywords:** pangasius, *Bacillus subtilis*, *Edwardsiella tarda*, probiotic, postbiotic, immune response, proinflammatory activity, antioxidative activity, growth performance

## Abstract

Edwardsiellosis, caused by *Edwardsiella tarda*, poses a significant threat to the aquaculture industry, particularly in pangasius farming. This study investigates the effects of probiotic *Bacillus subtilis* AAHM-BS2360 and its postbiotic metabolites on growth performance, immune responses, antioxidative activity, and disease resistance against *E. tarda* infection. A total of 240 healthy pangasius (37.0 ± 4.9 g) were divided into four treatment groups with four replicate tanks each, as follows: (1) the Control group, which received feed top-dressed with 100 mL of 0.85% NaCl/kg diet; (2) the Probiotic group, which received feed supplemented with 100 mL of *B. subtilis* AAHM-BS2360 cells at the concentration of 1 × 10^12^ CFU/kg diet; (3) the Postbiotic group, which received feed supplemented with *B. subtilis* AAHM-BS2360 cell-free supernatant 100 mL/kg diet; and (4) the Pro + Post group, which received a combination of *B. subtilis* AAHM-BS2360 cells and cell-free supernatant. After 30 days of feeding treatment, biochemical serum analysis revealed a significant increase in the AST/ALT ratio in the Postbiotic group. The Probiotic and Postbiotic treatments increased lysozyme activity in mucus, indicating an innate immune response to pathogens. The Pro + Post group exhibited the highest levels of catalase (CAT) in serum and upregulated antioxidant-related genes. All treatment groups receiving *B. subtilis* AAHM-BS2360, metabolites, and their combinations showed significant upregulation of immune-related genes, like *lygl1*, *tgfb*, *b2ml*, and *tnf.* The expression of proinflammatory genes (*litaf*, *ifngr1l*, *c3*, *il13*, and *il1b*) increased, with the most pronounced effects observed in the Pro + Post group. The Probiotic group showed significant upregulation of the growth-related gene *igf1*. Meanwhile, the Pro + Post group showed significantly higher values in SGR and ADG parameters, with values of 3.29 ± 0.98%/day and 1.42 ± 0.52 g/day respectively (*p* < 0.05). Survival rates were significantly higher in the Pro + Post (87.5%), Postbiotic (84.37%), and Probiotic (81.25%) groups when challenged with *E. tarda*. Dietary supplementation with *B. subtilis* AAHM-BS2360, its metabolites, and their combination enhanced immune response, reduced oxidative stress, and improved growth performance in pangasius, highlighting its potential as a functional feed additive for sustainable aquaculture.

## 1. Introduction

*Pangasianodon hypophthalmus*, commonly known as striped catfish, iridescent shark catfish, and pangasius, holds a prominent position in the global whitefish market. It contributes over 3.3 million tons annually and a value of USD 4.03 billion in 2022. Pangasius production is expected to grow up to 66.7% by 2030 to meet global demand, an increasing trend starting from December 2023, where the level of consumer confidence index in the United States for seafood products increased by 2.3%, as well as China’s growing market demand by establishing cooperation with several major catfish production countries [1,2,3]. The increasing demand for pangasius is likely to exert significant pressure on the aquafeed industry, with forecasts predicting an annual growth rate of 30% [4]. These developments present opportunities for increased pangasius production and market expansion, leading to the establishment of a thriving domestic and export pangasius farming industry. Pangasius farming not only enhances sustainability and competitiveness in national aquaculture but also serves as a crucial livelihood strategy for millions of people worldwide, and accessibility to low-income communities contributes to food security [5,6].

Pangasius production’s rapid expansion has caused health challenges, especially infectious diseases like Edwardsiellosis caused by *Edwardsiella tarda*. *E. tarda* is a Gram-negative bacterium in the family Enterobacteriaceae that causes systemic infections like hemorrhagic septicaemia, white nodules on internal organs, and has been confirmed zoonotic to humans [7,8]. Mass mortality caused by *E. tarda* has been reported in several pangasius farms in Kerala and Andhra Pradesh, India [9,10]. The overuse of antibiotics beyond the recommended dosage has been identified as a contributing factor to the rising prevalence of antibiotic resistance in Pangasius, a phenomenon observed in Bangladesh and Indonesia [11,12].

An environmentally friendly approach is needed to enhance pangasius’ immune response against Edwardsiellosis. Functional feed additives like probiotics, prebiotics, postbiotics, and synbiotics maintain a healthy gut microbiome, producing antimicrobial compounds, stimulating growth, and strengthening disease resistance in aquatic organisms [13,14,15]. Recent studies have underscored the potential of *Bacillus* spp. in aquaculture by showcasing its ability to develop the immune system (nonspecific and specific immunity) and induce different types of cytokines, namely *tumor necrosis factor* (*tnf*), *interleukin* (*il6*, *il1b*, *il8*, *il10*), and *myd88* [16]. In addition to the utilization of viable microorganisms belonging to *Bacillus* spp., the metabolite components of these microorganisms have been shown to enhance gastrointestinal health, reduce inflammation, and act as antibacterial and antifungal agents. The composition of bioactive metabolites termed postbiotics encompasses cell-free supernatant (CFS), cell-free spent media (CFSM), short-chain fatty acids (SCFAs), vitamins, various amino acids, enzymes, exopolysaccharides, and bacterial lysates [17,18].

*Bacillus subtilis* AAHM-BS2360 isolates, derived from the intestinal tract of healthy pangasius, have been found to contain bioactive components, including viable microorganisms (probiotics) as well as inert substrates or cell-free supernatants (CFSs or postbiotics). These components provide various advantages, serving as natural enhancers for the health and production of pangasius. Despite promising findings on probiotics and postbiotics of *Bacillus* spp. in aquaculture, a comprehensive understanding of their impacts on pangasius growth, antioxidative responses, proinflammatory responses, and immunity against *E. tarda* infections is lacking. This study investigates the impact of administering probiotic *Bacillus subtilis* AAHM-BS2360 and its bioactive components on pangasius growth and immune defenses. It specifically examines bactericidal and lysozyme activity and evaluates fish health through serum biochemical analysis. Furthermore, the study analyzes growth performance and explores the expression of proinflammatory, stress antioxidant, and immune-related genes of treated fish. These novelties provide key insights into current advancements in aquaculture additives, helping to foster a modern and thriving aquaculture industry.

## 2. Materials and Methods

### 2.1. Ethical Statement

The aquatic animal experiments were conducted following the Ethical Principles and Guidelines for the Use of Animals outlined by the National Research Council of Thailand, which governs the care and use of animals for research purposes. This protocol received approval from the Animal Ethics Committee at Kasetsart University in Thailand (Approval ID: ACKU67-FIS-015) on 20 May 2024.

### 2.2. Pangasius Husbandry

A total of 1000 pangasius fingerlings, with an average weight of 3.0 ± 0.5 g, were purchased from a local hatchery in Nakhon Pathom, Thailand, and subsequently transported to 3000 L freshwater nursery tank at the Center of Excellence in Aquatic Animal Health Management (CE AAHM), Faculty of Fisheries, Kasetsart University, Bangkok, Thailand. During the nursery stage, pangasius fingerlings were fed Higade 9006T (Charoen Pokphand Foods PCL, Bangkok, Thailand) commercial floating pellets three times daily with a feeding rate of 8.0% of body weight. The pellets contained 42.0% crude protein, 6.0% fat, 3.0% fiber, and 10.0% moisture. After a three-week nursery stage, the pangasius was transitioned to CP 9920D feed (Charoen Pokphand Foods PCL, Thailand) thrice daily at 5% of their body weight. The fish feed has a nutritional composition of 25% crude protein, 3% fat, 8% fiber, and 12% moisture.

A total of 240 pangasius from the nursery tank, with an average weight of 37.0 ± 4.9 g, were equally distributed among sixteen experimental tanks of 200 L and maintained with full aeration. The water quality was closely monitored and controlled weekly to ensure its optimal level for fish, with pH value within the range of 7.56–7.66, dissolved oxygen levels within the range of 3–5 ppm, temperature within the range of 28.69–28.97 °C, alkalinity values within the range of 111.4–113.3 mg/L as CaCO_3_, and nitrite levels within the range of 0.09–0.12 ppm. The initial biomass and average weight per tank were recorded before the experiment.

### 2.3. Bacterial Cultivation and Preparation of Experimental Diets

The *Bacillus subtilis* AAHM-BS2360 was obtained and initially identified by the Centre of Excellence in Aquatic Animal Health Management (CE AAHM), Faculty of Fisheries, Kasetsart University, Thailand. Initially isolated from the intestinal tract of a healthy pangasius from an earthen pond, it demonstrates significant in vitro antagonistic activity against *E. tarda*.

*B. subtilis* AAHM-BS2360 was routinely cultivated in tryptic soy broth (TSB, Difco^TM^, Baltimore, MD, USA) at pH 7.4 and incubated at 37 °C for 18–24 h. Bacterial density was standardized to 1 × 10^12^ CFU/mL using a UV-Vis spectrophotometer (600 nm, Thermo Fisher Scientific, Waltham, MA, USA), reaching an absorbance value of 1.4 for the combination of Probiotics and Postbiotics (Pro + Post) group. The culture was centrifuged at 3500× *g* for 10 min to obtain two fractions: the cell-free supernatant or postbiotic metabolite (Postbiotic group) and the pellet (Probiotic group). The supernatant was directly used for diet supplementation, while the pellet was washed, resuspended in 0.85% NaCl with the same initial concentration of 1 × 10^12^ CFU/mL, and subsequently used to supplement the diets of the Probiotic treatment group.

The experimental diet was prepared daily by enriching the commercial feed (CP 9920D) following a completely randomized design with four treatment groups, each consisting of four replicates containing 15 fish per replicate (60 fish/group). The groups were as follows: (1) the Control group, fed with regular feed top-dressed with 100 mL of 0.85% NaCl/kg diet; (2) the Probiotic group, which received feed supplemented with 100 mL of *B. subtilis* AAHM-BS2360 cells at the concentration of 1 × 10^12^ CFU/kg diet; (3) the Postbiotic group, which received feed supplemented with *B. subtilis* AAHM-BS2360 cell-free supernatant 100 mL/kg diet; and (4) the Pro + Post group, which received feed supplemented with a combination of *B. subtilis* AAHM-BS2360 cells and cell-free supernatant. The feeding experiment was conducted for 30 days, with a feeding rate of 4% body weight.

### 2.4. Growth Performance

The fish growth rates were determined through the calculation of the average daily growth rate (ADG) and the specific growth rate (SGR). The fish samples were weighed on a two-digit electronic scale. Daily records of feed consumption were diligently maintained, allowing for the computation of the feed conversion ratio (FCR). The detailed formulas for these calculations were provided by Bunnoy et al. [19].

### 2.5. Sample Collection

Following thirty days of dietary treatment, samples of tissue, including gills, skin mucus, whole blood, intestine, liver, and head kidney, were collected from eight fish/group (two fish/tank). These tissues were preserved in TriZOL^TM^ reagent (Thermo Fisher Scientific, Waltham, MA, USA) for gene expression assays and stored at −80 °C. Additionally, samples of tissues were placed in 1.5 mL tubes for immune parameter and antioxidant analyses, and stored at −20 °C. The whole blood (without anticoagulant) was collected in 1.5 mL tubes and allowed to clot at ambient temperature. After clotting, serum was separated by centrifugation at 3500× *g* for 15 min, then stored at −80 °C for further analysis. Eight fish per group were also sampled 24 h after the challenge test (according to Section 2.11), with head kidney, intestine, and whole blood collected for gene expression analysis.

### 2.6. Parameter of Serum Biochemical

The instrument utilized for serum biochemical assay was the Pushkang veterinary coagulation and chemistry combo analyzer, model MSC100V (Zhejiang PushKang Biotechnology Co., Ltd., Shaoxing, China). The reagent panel employed was the veterinary general chemistry 23 panel (CAT. no VE60010). The serum samples were immediately analyzed for the measurement of 23 biochemical parameters: albumin (ALB), total protein (TP), alkaline phosphatase (ALP), alanine aminotransferase (ALT), aspartate aminotransferase (AST), gamma-glutamyl transferase (GGT), direct bilirubin (DBIL), total bilirubin (TBIL), globulin (GLB), indirect bilirubin (IBIL), urea, calcium (Ca), creatinine (Crea), cholesterol, glucose (GLU), amylase (AMY), total bile acids (TBA), creatine kinase (CK), total carbon dioxide (tCO2), inorganic phosphorus (P), albumin/globulin ratio (ALB/GLB), urea/creatinine ratio (Urea/Crea), and AST/ALT ratio (AST/ALT).

### 2.7. Humoral Innate Immune Responses Assays

#### 2.7.1. Bactericidal Activity

The bactericidal activity assay was performed according to the previously published report [20]. A virulent freshwater fish pathogen *E. tarda* was routinely cultured in TSB medium using the conditions described in Section 2.3. This pathogen was utilized to evaluate the bactericidal activity of the mucus and serum samples. The concentration of *E. tarda* in the bacterial suspension was adjusted to 1 × 10^5^ CFU/mL in sterile phosphate-buffered saline (PBS), pH 7.4, by measuring the absorbance at 600 nm. Subsequently, 40 μL of each sample was gently mixed with 10 μL of 1 × 10^5^ CFU/mL *E. tarda* at room temperature for 90 min, and the survival of *E. tarda* was determined by counting the number of colonies on trypticase soy agar (TSA) (Merck KGaA, Darmstadt, Germany) plates after 24 h. After the incubation period, the bacterial colonies were counted as presented on the TSA plates. The bactericidal activity was determined using the following formula: BA (%) = [(T0 − T24)/T0] × 100, where T0 is the total initial bacterial count and T24 is the number of bacteria remaining on the plate after 24 h.

#### 2.7.2. Lysozyme Activity

The lysozyme activity was quantified using the approach adapted from the methodology originally proposed by Parry et al. [21]. A solution *Micrococcus lysodeikticus* (Sigma-Aldrich, Darmstadt, Germany) was prepared at a concentration of 0.2 mg/mL in phosphate-buffered saline (PBS) with pH 6.2. Subsequently, 10 µL of fish serum and mucus were added to a flat-bottomed microtiter plate, followed by the addition of 250 µL of *M. lysodeikticus* solution to each well. The decrease in absorbance at 540 nm was measured using a microplate spectrophotometer reader (iMark™ Microplate Absorbance Reader, BIO-RAD, Hercules, CA, USA), followed by incubation of the plate at room temperature for a period of 0 and 5 min. The units of lysozyme activity were calculated, where one unit is defined as the quantity of enzyme required to reduce the absorbance by 0.001 per minute, as described by Sukhsangchan et al. [22].

### 2.8. Determination of Oxidative Stress and Antioxidant Status of Target Organs

The tissues selected for antioxidative analysis included the serum, liver, intestine, skin, and gills. Fifty milligrams of each organ was accurately weighed and combined with 1 mL of PBS (0.1 M, pH 7.4). The mixture was then homogenized and centrifuged at 1100× *g* for 10 min at 4 °C. The supernatant was carefully transferred to a microcentrifuge tube and stored at −20 °C to ensure optimal conditions for subsequent analysis.

#### 2.8.1. Measurement of Catalase (CAT) Activity

CAT activity was examined according to the method adapted from Maehly [23]. One hundred microliters of the sample supernatant was mixed with 2.5 mL of phosphate buffer (50 mM, pH 5.0) and 0.4 mL of hydrogen peroxide (5.9 mM). The change in absorbance at 240 nm was recorded using a spectrophotometer every 30 s for 2 min. CAT activity in the sample was calculated by comparing it to the activity of the CAT standard. The CAT activity in the sample was expressed as units per minute per milligram of protein.

#### 2.8.2. Measurement of Glutathione Peroxidase (GPx) Activity

The measurement of GPx enzyme activity was conducted following the method adapted from Mohandas et al. [24]. A reaction mixture was prepared by combining the following components: 100 µL of sample supernatant, 1.49 mL of 0.1 M phosphate buffer (pH 7.4), 100 µL of 1 mM EDTA, 100 µL of 1 mM sodium azide, 50 µL of glutathione reductase (1 U/mL), 50 µL of 1 mM glutathione, 100 µL of 0.2 mM NADPH, and 10 µL of 0.25 mM H_2_O_2_. GPx activity was measured by monitoring the oxidation of NADPH at a wavelength of 340 nm at 25 °C. Enzymatic activity was expressed as µM of NADPH oxidized per minute per milligram (min^−1^mg^−1^) of protein, with a molar extinction coefficient of 6.22 × 10^3^ M^−1^ cm^−1^.

#### 2.8.3. Measurement of Glutathione Reductase (GR) Enzyme Activity

The activity of the GR enzyme was measured using the method modified by Sahreen et al. [25]. A reaction mixture was prepared by combining 100 µL of the sample supernatant with 100 µL of phosphate buffer (0.1 M, pH 7.6), 100 µL of oxidized glutathione (1 mM), and 100 µL of NADPH (0.1 mM). The mixture was incubated at 25 °C, and the absorbance was recorded at 340 nm. The GR enzyme activity was expressed as µM of NADPH oxidized min^−1^mg^−1^ of protein, calculated using a molar extinction coefficient of 6.22 × 10^3^ M^−1^ cm^−1^.

#### 2.8.4. Measurement of Reduced Glutathione (GSH) Content

The glutathione (GSH) content was determined using the method adapted from Jollow et al. [26]. The reaction mixture consisted of 1 mL of 4% *w*/*v* sulfosalicylic acid and 1 mL of the sample supernatant. After mixing thoroughly, the mixture was incubated at 4 °C for 1 h. Following incubation, the mixture was centrifuged at 1100× *g* at 4 °C for 20 min, and the supernatant was collected. A 100 µL aliquot of the supernatant was then mixed with 2.9 mL of a reaction solution containing 100 mM phosphate buffer (pH 7.4) and 100 mM 5,5′-dithiobis (2-nitrobenzoic acid) (DTNB). The absorbance of the resulting yellow chromogen was determined at 412 nm. A calibration curve for GSH was then generated, and the GSH content in the sample was expressed as nmol/mg of protein.

#### 2.8.5. Measurement of Glutathione-S-Transferase (GST) Activity

GST activity was assessed using the method described by Habig et al. [27]. A reaction mixture was prepared by combining 300 µL of the sample supernatant with 1.475 mL of 0.1 M phosphate buffer (pH 6.5), 200 µL of reduced glutathione (1 mM), and 25 µL of 1-Chloro-2,4-dinitrobenzene (CDNB) (1 mM). The absorbance of the resulting mixture at 340 nm was measured. Enzyme activity was calculated as nmol of CDNB conjugate in units per minute per milligram of protein, with a molar extinction coefficient of 9.6 × 10^3^ M^−1^ cm^−1^.

#### 2.8.6. Measurement of Malondialdehyde (MDA) Content

The MDA content in the samples was assessed using the thiobarbituric acid reactive substances (TBARS) assay, as described in the previous studies [28,29]. A 100 µL aliquot of the sample supernatant was mixed with 450 µL normal saline solution (0.85% *w*/*v*), 1000 µL of trichloroacetic acid (TCA, 10% *w*/*v*), and 200 µL of thiobarbituric acid (TBA, 0.67% *w*/*v*). The reaction mixture was boiled for 30 min and cooled. Subsequently, 2 mL of distilled water was added, and the mixture was centrifuged at 1100× *g* for 10 min. The supernatant was collected, and the absorbance was measured using a spectrophotometer at 532 nm. The resulting value was used to calculate the MDA content by comparing it with a standard curve of tetramethoxypropane (TMP). The MDA amount in the sample is expressed as nmol/mg of protein.

#### 2.8.7. Measurement of Superoxide Dismutase (SOD) Activity

SOD activity was measured using the modified method obtained from Takada et al. [29,30]. A 100 µL aliquot of the sample supernatant was mixed with 1 mL of a solution containing 0.1 mM xanthine, 0.025 mM nitro blue tetrazolium salt, 0.1 mM disodium ethylenediaminetetraacetate dihydrate, 60 mM sodium carbonate buffer (pH 10.2), and xanthine oxidase. The change in absorbance at 560 nm was measured every 30 s for 2 min, and the calibration curve for the SOD standard was plotted. The SOD activity in the sample was expressed as units per minute per milligram of protein.

### 2.9. cDNA Preparation and Gene Expression Analysis Using Quantitative Reverse Transcription PCR (qRT-PCR)

Total RNA was extracted from head kidney, gills, intestine, whole blood, liver, and spleen samples according to TriZOL^TM^ manual protocol (Thermo Fisher Scientific, Waltham, MA, USA). The total RNA was quantified using a NanoDrop™ spectrophotometer (Thermo Fisher Scientific, Waltham, MA, USA). A total of 1 µg of total RNA was used as a template to synthesize first-strand complementary DNAs (cDNAs) using Maxime™ RT PreMix (iNtRON Biotechnology, Seongnam-si, Republic of Korea). The synthesized first-strand cDNA was stored at −20 °C for further analysis.

The quantitative real-time PCR (qRT-PCR) assay was performed using 2× RealMOD^TM^ Green W^2^ 2× qPCR Mix-SYBR^®^ (iNTRON Biotechnology, Republic of Korea) in an Azure Cielo^TM^ real-time PCR (Azure biosystems, Dublin, CA, USA). The qPCR cycling conditions consisted of an initial condition of one cycle of 95 °C for 5 min, 40 cycles of 95 °C for 30 s, 60 °C for 30 s, 72 °C for 90 s, and a final extension at 72 °C for 10 min. The housekeeping genes, including *actb1* and *rna18s*, were used to standardize mRNA and cDNA quantities, to ensure consistency and reliability of the results. The target genes were analyzed using qRT-PCR to assess non-specific immune-related genes, proinflammatory genes, antioxidant-related genes, and growth-related genes.

All primers were standardized to ensure amplification efficiency before the qRT-PCR analysis. The relative expression levels of genes in the fish tissues were calculated using the 2^−ΔΔCT^ analysis according to the protocol of Livak and Schmittgen [31]. The primer sequences of all targeted genes are detailed in Table 1.

### 2.10. Disease Resistance and Survival Rate (SR) After E. tarda Challenges

At the end of the experiment, 40 fish were randomly selected from each group (10 fish per replicate) and transferred to 250 L fiberglass tanks with full aeration. These fish were then subjected to intraperitoneal (*i.p*) injection with *E. tarda*. The cultivation of *E. tarda* was routinely carried out in TSB, and the concentration was adjusted using a spectrophotometer as described in Section 2.3.

Each fish was injected with a 100 μL solution containing *E. tarda* at a concentration of 1 × 10^8^ CFU/mL (1 × 10^7^ CFU/fish). The optimum concentrations and dosages were determined based on preliminary 14-day LD_50_ tests conducted on the fish. At 24 h post-infection, eight surviving fish from each group were selected for sampling gene expression parameters as described in the previous section. Daily mortality rates were recorded for 14 days, and the survival rate (SR) was analyzed using the Kaplan–Meier method [32].

### 2.11. Statistical Data Analysis

The statistical analysis was performed using GraphPad Prism 10 version 10.4.1 for macOS (GraphPad Software, Boston, MA, USA), employing one-way analysis of variance (ANOVA) followed by Tukey’s multiple comparison test after passing the Shapiro–Wilk test to assess the normality of data distribution. Survival curves were created using GraphPad Prism 10 to visualize the survival rate profile over time. Data are shown as means ± standard deviations (SD). Letter superscripts and asterisks (*) indicate statistical significance differences among groups, with a threshold of *p* < 0.05. Statistical significance was determined at *p*-values of * (*p* < 0.05), ** (*p* < 0.01), and *** (*p* < 0.001).

## 3. Results

### 3.1. Serum Biochemical Analysis

**Figure 1:**
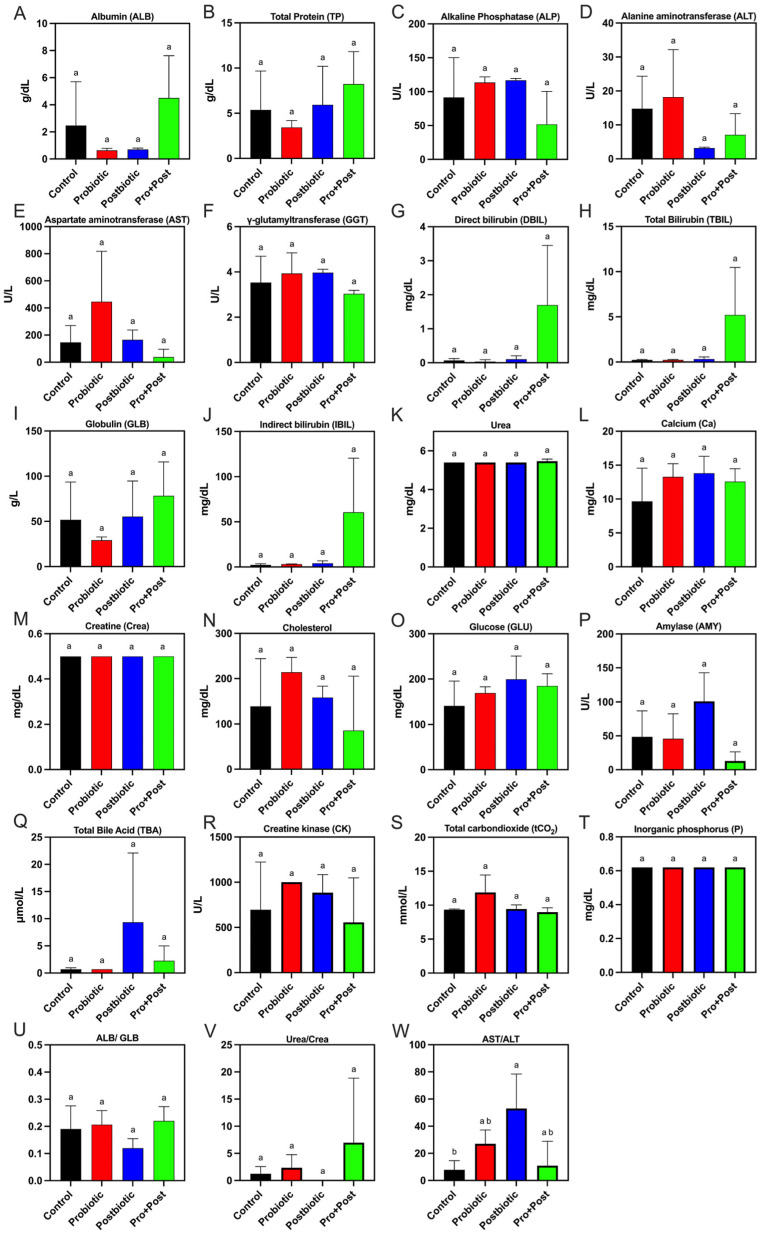
(**A**–**W**) Biochemistry of *P. hypophthalmus* following 30-day supplementation with probiotic, postbiotic, and their combination derived from *B. subtilis* AAHM-BS2360. Data are represented as mean ± SD (n = 8). Data in each graph with different letters denote statistically significant differences (*p* < 0.05).

Most serum biochemical analysis results showed non-significant values for all treatments across all biochemical test parameters (*p* > 0.05), except for the AST/ALT ratio parameter, which exhibited significant values in the postbiotic *B. subtilis* AAHM-BS2360 treatment compared to the control (*p* < 0.05). The serum biochemical values are presented in Figure 1A–W.

The albumin parameters in all treatment groups exhibited mean values ranging from 0.633 to 4.5 g/dL, while the globulin levels demonstrated mean values from 29.17 to 78.33 g/L. The ALB/GLB ratio attained a mean value of 0.18. The mean concentrations of bilirubin in serum samples, comprising DBIL, IBIL, and TBIL, were found to be 0.475 mg/dL, 1.499 mg/dL, and 17.541 mg/dL, respectively. Furthermore, a similar pattern was observed across all treatments for the parameters of urea, creatine, and inorganic phosphorus, with values of 5.415 mg/dL, 0.5 mg/dL, and 0.62 mg/dL, respectively. The urea/creatine ratio obtained a mean value of 2.65.

The mean value of total protein parameters ranged from 3.43 g/dL to 8.23 g/dL, while the GGT content demonstrated a mean value of 3.61 U/L. The tCO_2_ levels exhibited an average value of 9.9 mmol/L, and alkaline phosphatase levels exhibited a range of 51.67–116.8 U/L. The cholesterol and glucose levels in serum samples exhibited an average range of 85.70–214.0 mg/dL and 141.1–199.3 mg/dL, respectively. Calcium and amylase levels demonstrated mean values of 12.32 mg/dL and 52.01 U/L.

The range of mean values of total bile acid levels across all treatments was found to be between 0.7 and 9.3 µmol/L, while the mean value of creatine kinase was recorded as 783.9 U/L. Furthermore, the range of mean values of ALT and AST in all treatments was determined to be 3.167–18.2 U/L and 37.5–446.6 U/L, respectively. The AST/ALT ratio exhibited a range of mean values from 7.9 to 53.0, indicating that Postbiotic treatment resulted in a significant increase compared to the control treatment (*p* < 0.05).

### 3.2. Nonspecific Humoral Immune Responses

#### 3.2.1. Bactericidal Activity

**Figure 2:**
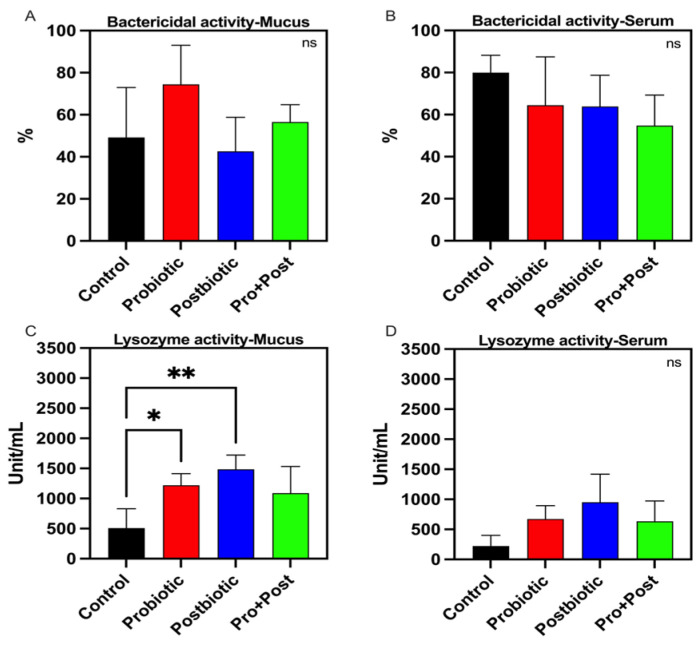
Bactericidal activity (%) response in mucus (**A**) and serum (**B**), and lysozyme activity (Unit/mL) in mucus (**C**) and serum (**D**) of *P. hypophthalmus* following 30-day supplementation with probiotic, postbiotic, and their combination derived from *B. subtilis* AAHM-BS2360. Data are represented as mean ± SD (n = 8). Asterisks indicate statistically significant differences (ns = not significant). * (*p* < 0.05), ** (*p* < 0.01).

The investigation revealed no statistically significant differences in bactericidal activity parameters between the serum and mucus samples among groups (*p* > 0.05). The bactericidal activity values for each group are presented in Figure 2A,B.

#### 3.2.2. Lysozyme Activity

Statistical analysis of lysozyme activity in mucus samples revealed a substantial increase in both Probiotic and Postbiotic treatments in comparison to the Control group (*p* < 0.05; *p* < 0.01), with values recorded at 1220 ± 194.1 units/mL and 1487 ± 234.7 units/mL, respectively, as depicted in Figure 2C. The serum samples exhibited comparable lysozyme activity parameters across all treatment groups (*p* > 0.05), as illustrated in Figure 2D.

### 3.3. Responses of Oxidative Stress and Antioxidant Activity

#### 3.3.1. Liver

**Figure 3:**
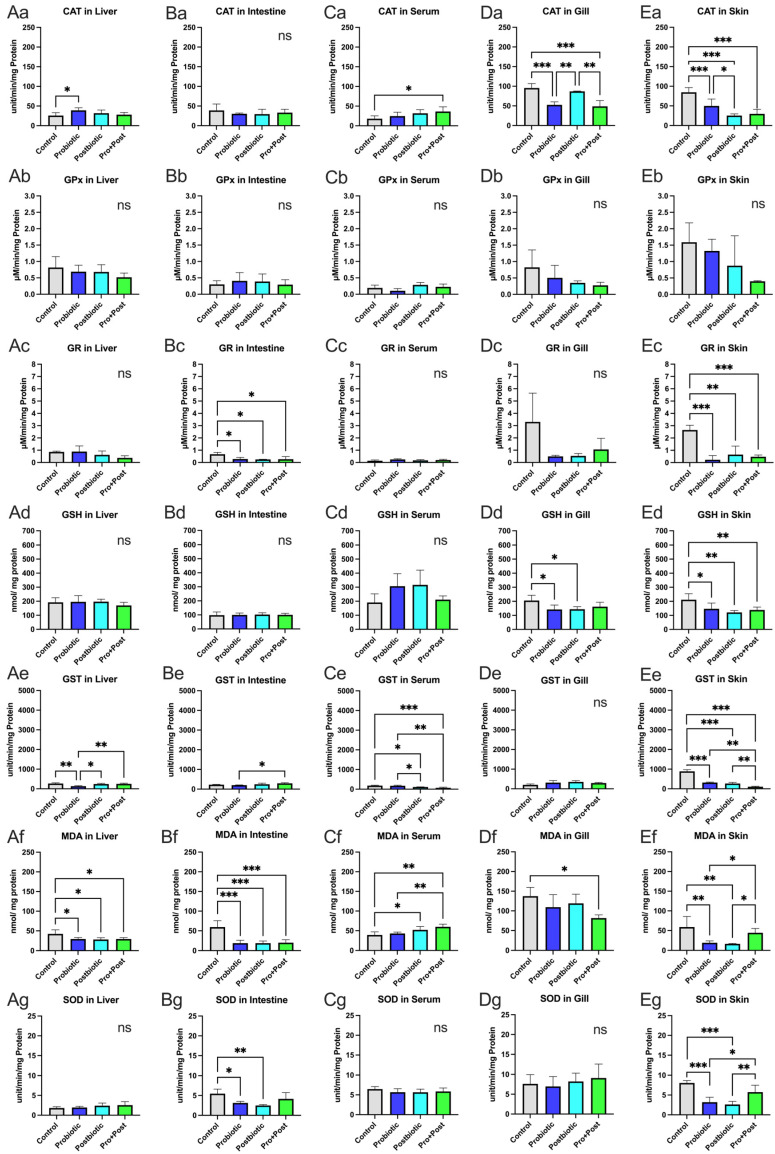
Levels of MDA and GSH, and activities of CAT, SOD, GPx, GR, and GST in liver (**Aa**–**Ag**), intestine (**Ba**–**Bg**), serum (**Ca**–**Cg**), gills (**Da**–**Dg**), and skin (**Ea**–**Eg**) of *P. hypophthalmus* following 30-day supplementation with probiotic, postbiotic, and their combination derived from *B. subtilis* AAHM-BS2360. Data are represented as mean ± SD (n = 8). Asterisks indicate statistically significant differences (ns = not significant). * (*p* < 0.05), ** (*p* < 0.01), and *** (*p* < 0.001).

The GPx, GR, and SOD activities and the GSH level in the liver showed no significant differences among the treatments (*p* > 0.05). CAT activity was significantly higher in the Probiotic group compared to the Control (*p* < 0.05), with a peak of 39.12 ± 6.45 units/min/mg protein. The Control group had the highest GST and MDA levels. GST was significantly lower in the Probiotic group compared to the Control, Postbiotic, and Pro + Post groups (*p* < 0.01; *p* < 0.05). All *B. subtilis* AAHM-BS2360-treated groups showed significant reduction in MDA levels compared to the Control group (*p* < 0.05). The graphical details are presented in Figure 3(Aa–Ag).

#### 3.3.2. Intestine

The MDA levels in the intestine were highest in the Control group (59.28 ± 16.37 nmol/mg protein) and significantly differed from all treatments (*p* < 0.001). The GR and SOD levels were also highest in the Control group, with significant differences from other groups (*p* < 0.05; *p* < 0.01). The Pro + Post group showed a significant increase in GST activity (294.1 ± 31.26 units/min/mg protein) compared to the Probiotic group (*p* < 0.05). No significant differences were found in CAT and GPx activities and GSH levels across the treatments (*p* > 0.05) (Figure 3(Ba–Bg)).

#### 3.3.3. Serum

The GPx, GR, and SOD activities and GSH level showed no significant differences among groups (*p* > 0.05). The Pro + Post group showed the highest CAT activity and MDA level (36.3 ± 11.93 units/min/mg protein; 59.97 ± 6.68 nmol/mg protein), significantly higher than the Control (*p* < 0.05; *p* < 0.01) and Probiotic groups (*p* < 0.01). The Postbiotic group also showed higher MDA level than the Control group (*p* < 0.05). For GST, the Control group had significantly higher values than the Postbiotic and Pro + Post groups (*p* < 0.05; *p* < 0.001), while the Probiotic group showed the highest GST activity (173.5 ± 29.31 nM/min/mg protein), significantly exceeding Postbiotic and Pro + Post treatments (*p* < 0.05; *p* < 0.01) (Figure 3(Ca–Cg)).

#### 3.3.4. Gills

The Control group showed the highest CAT activity (95.43 ± 11.31 units/min/mg protein), significantly exceeding those in the Probiotic and Pro + Post groups (*p* < 0.001). The Postbiotic group also had significantly higher CAT activity than the Probiotic and Pro + Post groups (*p* < 0.01). GSH levels in the Control group differed significantly from the Probiotic and Postbiotic groups (*p* < 0.05), while MDA was significantly higher than in the Pro + Post group (*p* < 0.05). No significant differences were found in GPx, GR, GST, and SOD across the treatments (*p* > 0.05) (Figure 3(Da–Dg)).

#### 3.3.5. Skin

Oxidative status in the skin showed that the Control group had the highest values for most parameters, except GPx, which showed no significant differences across the treatments (0.39–1.58 µM/min/mg protein; *p* > 0.05). CAT activities were significantly higher in the Control group compared to all groups (*p* < 0.001), with the Probiotic group also higher than the Postbiotic group (*p* < 0.05).

GR activity and GSH level followed a similar trend, with the Control group significantly higher than all other treatments (*p* < 0.05; *p* < 0.01; *p* < 0.001). For GST, the Control group (888.2 ± 86.23 nM/min/mg protein) was significantly higher than all *B. subtilis* AAHM-BS2360-treated groups (*p* < 0.001), and the Probiotic and Postbiotic groups differed from the Pro + Post group (*p* < 0.01). In MDA and SOD, the Control and Pro + Post group showed significantly higher values than the Probiotic and Postbiotic groups (*p* < 0.05; *p* < 0.01; *p* < 0.001) (Figure 3(Ea–Eg)).

### 3.4. Oxidative Stress and Antioxidant-Related Gene Expression

#### 3.4.1. Following a 30-Day Supplementation with Probiotic, Postbiotic, and Their Combination Derived from *B. subtilis* AAHM-BS2360

**Figure 4:**
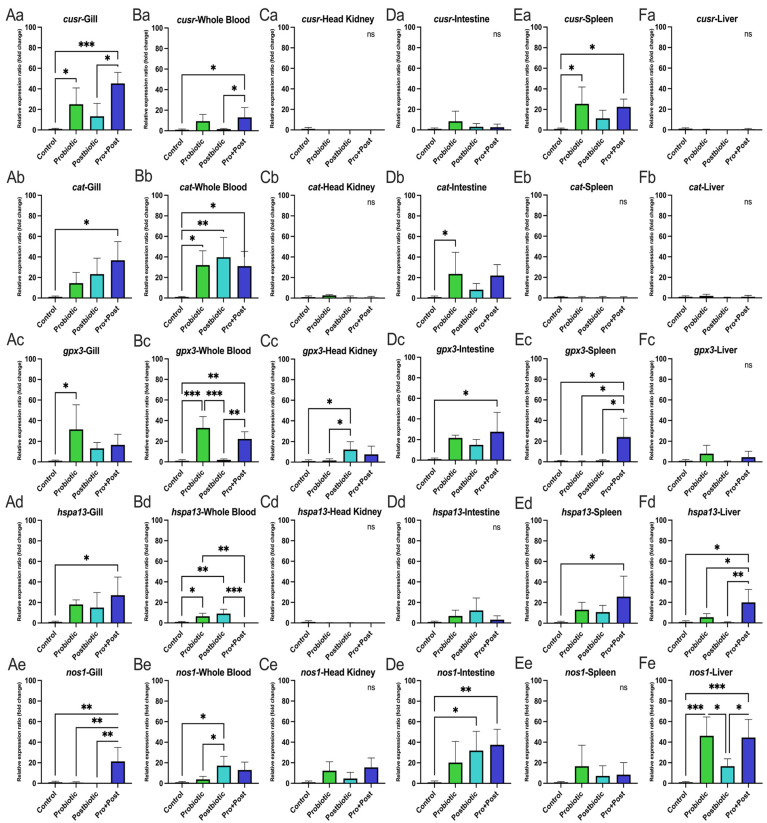
Relative stress oxidative and antioxidant-related gene expression (fold change) including *cusr*, *cat*, *gpx3*, *hspa13*, and *nos1* in gills (**Aa**–**Ae**), whole blood (**Ba**–**Be**), head kidney (**Ca**–**Ce**), intestine (**Da**–**De**), spleen (**Ea**–**Ee**), and liver (**Fa**–**Fe**) of *P. hypophthalmus* following 30-day supplementation with probiotic, postbiotic, and their combination derived from *B. subtilis* AAHM-BS2360. Data are represented as mean ± SD (n = 8). Asterisks indicate statistically significant differences (ns = not significant). * (*p* < 0.05), ** (*p* < 0.01), and *** (*p* < 0.001).

The Pro + Post group showed significant upregulation of *cusr* gene expression in the gill and whole blood compared to the Control and Postbiotic group (*p* < 0.05; *p* < 0.001), as well as significantly higher in the spleen compared to the Control group (*p* < 0.05). The Probiotic group showed increased *cusr* expression in the gills and spleen compared to the Control group (*p* < 0.05) (Figure 4(Aa,Ba,Ea)). No significant differences were observed in the head kidney, intestine, or liver across treatments (*p* > 0.05) (Figure 4(Ca,Da,Fa)).

In whole blood, all *B. subtilis* AAHM-BS2360-based treatments (Probiotic, Postbiotic, Pro + Post) showed higher *cat* expression compared to the Control group (*p* < 0.05; *p* < 0.01). In the gills, the Pro + Post group showed a significant increase of *cat* gene over the Control (*p* < 0.05), while in the intestine, the Probiotic group exhibited significant upregulation compared to the Control group (*p* < 0.05) (Figure 4(Ab,Bb,Db)). No significant differences were observed in the head kidney, spleen, or liver (*p* > 0.05) (Figure 4(Cb,Eb,Fb)).

Expression of the antioxidant-related *gpx3* gene showed no significant differences in the liver across treatments (*p* > 0.05) (Figure 4(Fc)). However, the Probiotic group showed significant *gpx3* upregulation in the gills (*p* < 0.05) compared to the Control group and in whole blood compared to the Control and Postbiotic groups (*p* < 0.001). The Pro + Post group also showed higher *gpx3* expression in whole blood compared to the Postbiotic and Control groups (*p* < 0.01). In the head kidney, the Postbiotic group had significantly higher *gpx3* expression than the Control and Probiotic groups (*p* < 0.05). The Pro + Post group showed elevated *gpx3* expression in the intestine and spleen compared to the Control group and other treatments, respectively (*p* < 0.05) (Figure 4(Ac,Bc,Cc,Dc,Ec)).

The Pro + Post group showed significant upregulation of the *hspa13* gene in the gill, spleen, and liver compared to the Control group (*p* < 0.05). In the liver, *hspa13* expression was also significantly higher in the Pro + Post group than in the Probiotic and Postbiotic groups (*p* < 0.05; *p* < 0.01). In whole blood, the Probiotic and Postbiotic groups had significantly higher *hspa13* expression than both the Control and Pro + Post groups (*p* < 0.05; *p* < 0.001) (Figure 4(Ad,Bd,Ed,Fd)). No significant differences were observed in the head kidney and intestine (*p* > 0.05) (Figure 4(Cd,Dd))

As shown in Figure 4(Ce,Ee), *nos1* expression showed no significant differences in the head kidney and spleen across treatments (*p* > 0.05). In the gills, the Pro + Post group showed significantly higher *nos1* expression than all other groups (*p* < 0.01). In whole blood, the Postbiotic group had the highest *nos1* expression, significantly exceeding the Control and Probiotic groups (*p* < 0.05). In the intestine, both Pro + Post and Postbiotic groups showed elevated *nos1* expression compared to the Control group (*p* < 0.01; *p* < 0.05). In the liver, *nos1* was significantly upregulated in the Probiotic and Pro + Post groups compared to the Control and Postbiotic groups (*p* < 0.05; *p* < 0.001) (Figure 4(Ae,Be,De,Fe)).

#### 3.4.2. Post-Challenge with *E. tarda*

**Figure 5:**
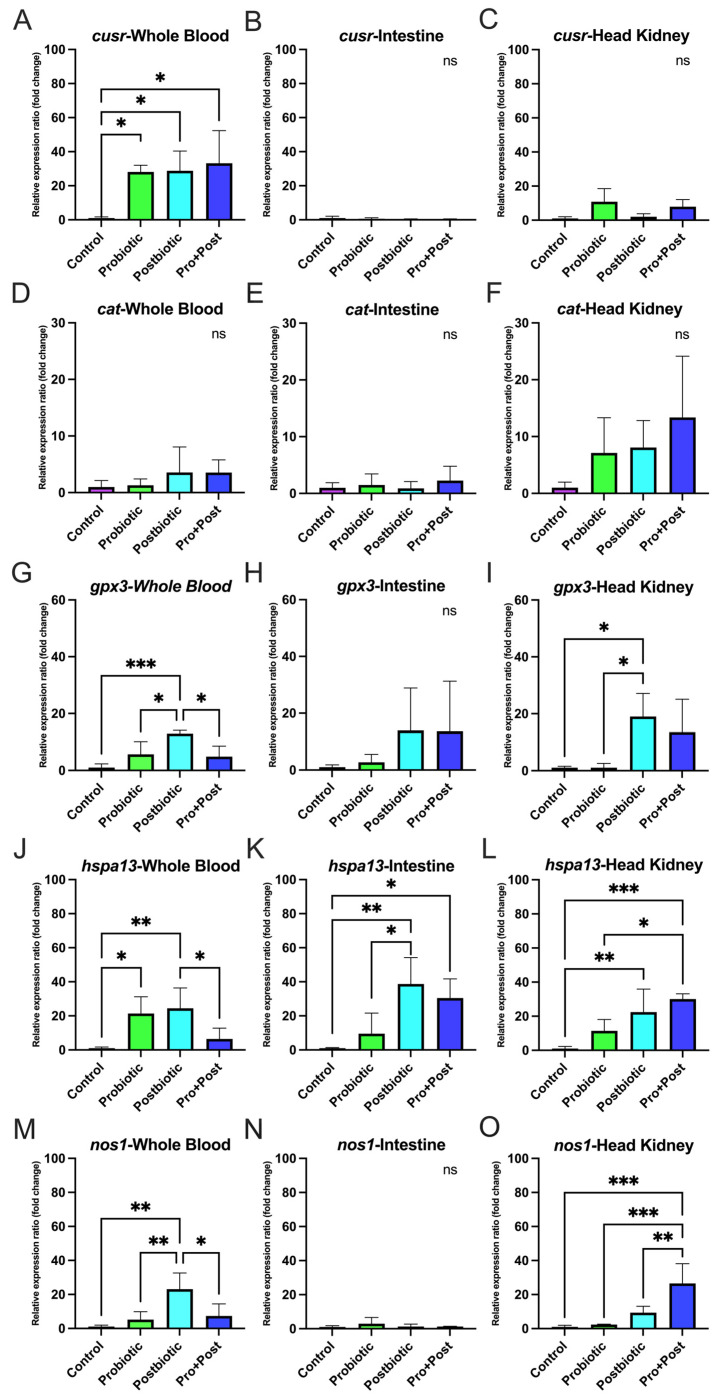
Relative antioxidant-related gene expression (fold change) including *cusr* (**A**–**C**), *cat* (**D**–**F**), *gpx3* (**G**–**I**), *hspa13* (**J**–**L**), and *nos1* (**M**–**O**) in whole blood, intestine, and head kidney of *P. hypophthalmus* following 30-day supplementation with probiotic, postbiotic, and their combination derived from *B. subtilis* AAHM-BS2360 after being challenged with *E. tarda*. Data are represented as mean ± SD (n = 8). Asterisks indicate statistically significant differences (ns = not significant). * (*p* < 0.05), ** (*p* < 0.01), and *** (*p* < 0.001).

In whole blood, *cusr* expression was significantly higher in all treated groups compared to the Control group (*p* < 0.05). Similarly, *gpx3* and *nos1* were significantly upregulated in the Postbiotic group compared to all other treatments (*p* < 0.05; *p* < 0.01; *p* < 0.001). For *hspa13*, the Postbiotic group showed significantly higher expression than the Pro + Post and Control groups, while the Probiotic group also showed increased *hspa13* expression compared to the Control group (*p* < 0.05; *p* < 0.01) (Figure 5A,G,J,M). Expression of the *cat* gene showed no significant differences among treatments (*p* > 0.05) (Figure 5D).

In the intestine, only the *hspa13* gene was significantly upregulated in the Postbiotic group compared to the Control and Probiotic groups (*p* < 0.05; *p* < 0.01), while the Pro + Post group also showed higher *hspa13* expression than Control (*p* < 0.05) (Figure 5K). No significant differences were observed in *cusr*, *cat*, *gpx3*, or *nos1* expression in the intestine across treatments (*p* > 0.05) (Figure 5B,E,H,N).

In the head kidney, *cusr* and *cat* gene expression showed no significant differences among treatments (*p* > 0.05) (Figure 5C,F). However, the *gpx3* gene was significantly upregulated in the Postbiotic group compared to the Probiotic and Control groups (*p* < 0.05). Both Postbiotic and Pro + Post groups showed increased *hspa13* expression compared to the Control group (*p* < 0.01; *p* < 0.001), with the Pro + Post group also differing significantly from the Probiotic group (*p* < 0.01). Notably, *nos1* expression was significantly elevated in the Pro + Post group compared to all other treatments (*p* < 0.01, *p* < 0.001) (Figure 5I,L,O).

### 3.5. Non-Specific Immune-Related Gene Expression

#### 3.5.1. Following a 30-Day Supplementation with Probiotic, Postbiotic, and Their Combination Derived from *B. subtilis* AAHM-BS2360

**Figure 6:**
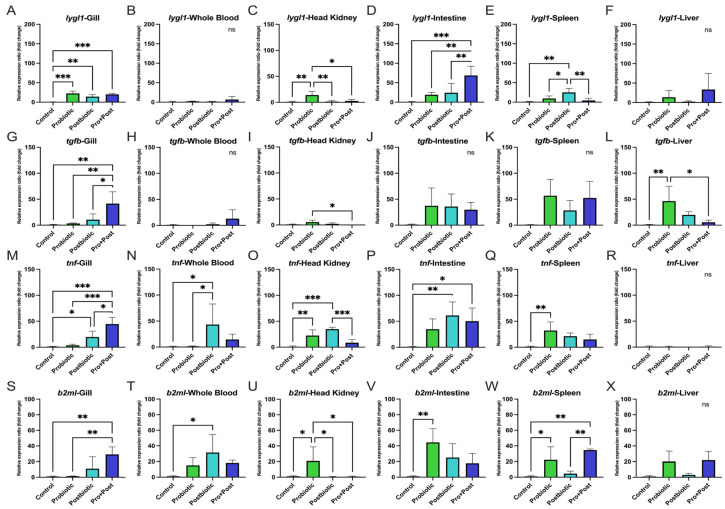
Relative immune-related gene expression (fold change) including *lygl1* (**A**–**F**), *tgfb* (**G**–**L**), *tnf* (**M**–**R**), and *b2ml* (**S**–**X**) in gill, whole blood, head kidney, intestine, spleen, and liver of *P. hypophthalmus* following 30-day supplementation with probiotic, postbiotic, and their combination derived from *B. subtilis* AAHM-BS2360. Data are represented as mean ± SD (n = 8). Asterisks indicate statistically significant differences (ns = not significant). * (*p* < 0.05), ** (*p* < 0.01), and *** (*p* < 0.001).

All treatments containing *B. subtilis* AAHM-BS2360 significantly upregulated *lygl1* genes in the gills compared to the Control group (*p* < 0.01; *p* < 0.001). The Pro + Post group showed the strongest activation of *tgfb* and *tnf* compared to all treatment groups (*p* < 0.05; *p* < 0.01; *p* < 0.001). For *b2ml*, the Pro + Post group showed significantly higher expression than the Control and Probiotic group (*p* < 0.01). The Postbiotic group also upregulated *tnf* gene compared to the Control group (*p* < 0.05) (Figure 6A,G,M,S).

No significant differences in *lygl1* expression were observed in whole blood and liver across treatments (*p* > 0.05). In the intestine, the Pro + Post group showed the highest *lygl1* expression compared to all groups (*p* < 0.01; *p* < 0.001). In the head kidney, the Probiotic group had the highest *lygl1* expression (*p* < 0.05; *p* < 0.01), while in the spleen, the Postbiotic group showed significantly elevated *lygl1* gene expression compared to all other treatments (*p* < 0.05; *p* < 0.01) (Figure 6B–F).

For the *tgfb* gene, the Probiotic group showed significantly higher expression in the head kidney compared to the Pro + Post group (*p* < 0.05). A similar pattern was seen in the liver, where *tgfb* expression was higher in the Probiotic group than in the Control and Pro + Post groups (*p* < 0.01; *p* < 0.05). No significant differences were observed in whole blood, intestine, or spleen across treatments (*p* > 0.05) (Figure 6I–L).

In the liver, no significant differences were found in *tnf* and *b2ml* expression across treatments (*p* > 0.05) (Figure 6R,X). The Postbiotic group showed significant upregulation of *tnf* in whole blood, head kidney, and intestine compared to the Control group (*p* < 0.05; *p* < 0.01; *p* < 0.001), and exceeded the Probiotic group in whole blood (*p* < 0.05) and the Pro + Post group in the head kidney (*p* < 0.001). The Probiotic and Pro + Post groups showed significant upregulation of *tnf* gene in head kidney, spleen, and intestine compared to each Control group, respectively (*p* < 0.01; *p* < 0.05) (Figure 6N–Q).

The *b2ml* gene expression was significantly higher in the Postbiotic group compared to the Control group in whole blood (*p* < 0.05). The Probiotic group showed upregulated *b2ml* in the head kidney and intestine compared to the Control group (*p* < 0.05; *p* < 0.01) and was also significantly higher than both Postbiotic and Pro + Post groups in the head kidney (*p* < 0.05). In the spleen, *b2ml* was significantly upregulated in the Probiotic group compared to the Control group (*p* < 0.05), while the Pro + Post group showed the highest *b2ml* expression, significantly exceeding the Control and Postbiotic groups (*p* < 0.01) (Figure 6T–W).

#### 3.5.2. Post-Challenge with *E. tarda*

**Figure 7:**
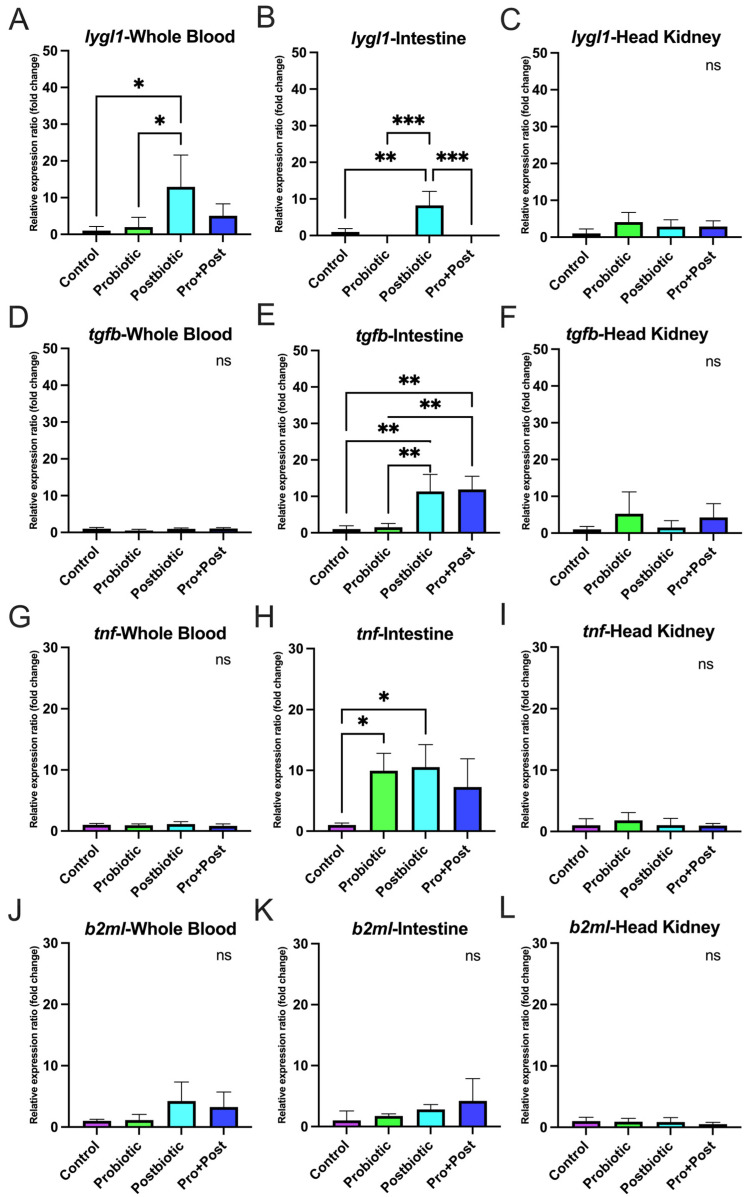
Relative immune-related gene expression (fold change) including *lygl1* (**A**–**C**), *tgfb* (**D**–**F**), *tnf* (**G**–**I**), and *b2ml* (**J**–**L**) in head kidney, intestine, and whole blood of *P. hypophthalmus* following 30-day supplementation with probiotic, postbiotic, and their combination derived from *B. subtilis* AAHM-BS2360 after being challenged with *E. tarda*. Data are represented as mean ± SD (n = 8). Asterisks indicate statistically significant differences (ns = not significant). * (*p* < 0.05), ** (*p* < 0.01), and *** (*p* < 0.001).

In whole blood, *lygl1* expression was significantly higher in the Postbiotic group compared to the Control and Probiotic groups (*p* < 0.05), while *tgfb*, *tnf*, and *b2ml* showed no significant differences among treatments (Figure 7A,D,G,J).

In the intestine, *lygl1* expression was highest in the Postbiotic group, significantly exceeding all other treatments (*p* < 0.01; *p* < 0.001). The *tgfb* expression was significantly elevated in both the Postbiotic and Pro + Post groups compared to the Control and Probiotic groups (*p* < 0.01). The *tnf* gene expression was also significantly upregulated in the Probiotic and Postbiotic groups compared to the Control group (*p* < 0.05). However, *b2ml* expression showed no significant differences among treatments (*p* > 0.05) (Figure 7B,E,H,K). In the head kidney, no significant changes were observed in any immune gene expression across treatment groups (*p* > 0.05) (Figure 7C,F,I,L).

### 3.6. Proinflammatory-Related Gene Expression

#### 3.6.1. Following a 30-Day Supplementation with Probiotic, Postbiotic, and Their Combination Derived from *B. subtilis* AAHM-BS2360

**Figure 8:**
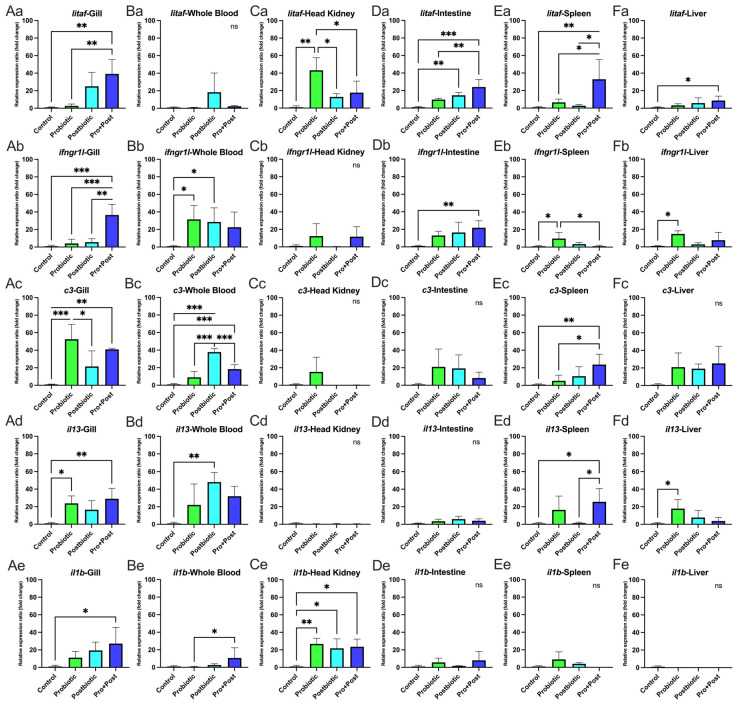
Relative proinflammatory-related gene expression (fold change), including *litaf*, *ifngr1l*, *c3*, *il13*, and *il1b* in gills (**Aa**–**Ae**), whole blood (**Ba**–**Be**), head kidney (**Ca**–**Ce**), intestine (**Da**–**De**), spleen (**Ea**–**Ee**), and liver (**Fa**–**Fe**) of *P. hypophthalmus* following 30-day supplementation with probiotic, postbiotic, and their combination derived from *B. subtilis* AAHM-BS2360. Data are represented as mean ± SD (n = 8). Asterisks indicate statistically significant differences (ns = not significant). * (*p* < 0.05), ** (*p* < 0.01), and *** (*p* < 0.001).

No significant differences in expression were observed for *litaf* in whole blood, *ifngr1l* in the head kidney, *c3* in the head kidney, intestine, and liver, *il13* in the head kidney and intestine, or *il1b* in the intestine, spleen, and liver (*p* > 0.05) (Figure 8(Ba,Cb–Cd,Dc–De,Ee,Fc,Fe)).

The Pro + Post group showed significantly higher *litaf* expression than the Control group in the gill, intestine, spleen, and liver (*p* < 0.05; *p* < 0.01; *p* < 0.001). Compared to the Probiotic group, *litaf* was also significantly upregulated in the Pro + Post group in the gill, intestine, and spleen (*p* < 0.05; *p* < 0.01). In the spleen, *litaf* expression in the Pro + Post group was significantly higher than in the Postbiotic group (*p* < 0.05). In the head kidney, the Probiotic group dominated significantly *litaf* expression compared to other groups (*p* < 0.05; *p* < 0.01), while in the intestine, the Postbiotic group exhibited substantially higher *litaf* expression than the Control group (*p* < 0.01). Detailed graphs of *litaf* gene are presented in Figure 8(Aa,Ca,Da,Ea,Fa).

The Pro + Post group showed significantly elevated *ifngr1l* expression in the gills compared to other groups (*p* < 0.01; *p* < 0.001) and in the intestine compared to the Control group (*p* < 0.01). The Probiotic group exhibited increased *ifngr1l* expression in whole blood, spleen, and liver to the Control group (*p* < 0.05) and showed higher expression in the spleen than the Pro + Post group (*p* < 0.05). Additionally, the Postbiotic group displayed significantly upregulated *ifngr1l* expression in whole blood compared to the Control (*p* < 0.05) (Figure 8(Ab,Bb,Db,Eb,Fb)).

In the gills, *c3* expression was significantly elevated in the Probiotic and Pro + Post groups compared to the Control group (*p* < 0.01; *p* < 0.001), with the Probiotic group also showing higher expression than the Postbiotic group (*p* < 0.05). In whole blood, the Postbiotic group exhibited the highest *c3* expression, surpassing to other treatments (*p* < 0.001), while the Pro + Post group also showed significantly higher expression than Control (*p* < 0.001). In the spleen, *c3* expression was significantly upregulated in the Pro + Post group compared to the Probiotic and Control groups (*p* < 0.05; *p* < 0.001) (Figure 8(Ac,Bc,Ec)).

The *il13* gene was significantly upregulated in the gills and liver of the Probiotic group compared to the Control group (*p* < 0.05). In the gills, the Pro + Post group also showed higher *il13* expression than the Control group (*p* < 0.01). In whole blood, the Postbiotic group had significantly elevated *il13* expression compared to the Control group (*p* < 0.01), while the Pro + Post group showed higher expression than both the Control and Postbiotic groups (*p* < 0.05) (Figure 8(Ad,Bd,Ed,Fd)).

In the gills, *il1b* expression was significantly upregulated in the Pro + Post group compared to the Control group (*p* < 0.05). In whole blood, *il1b* expression in the Pro + Post group differed significantly from the Probiotic group (*p* < 0.05). In the head kidney, all *B. subtilis* AAHM-BS2360-based treatments (Probiotic, Postbiotic, and Pro + Post) showed significantly higher *il1b* expression compared to the Control group (*p* < 0.05; *p* < 0.01) (Figure 8(Ae,Be,Ce)).

#### 3.6.2. Post-Challenge with *E. tarda*

**Figure 9:**
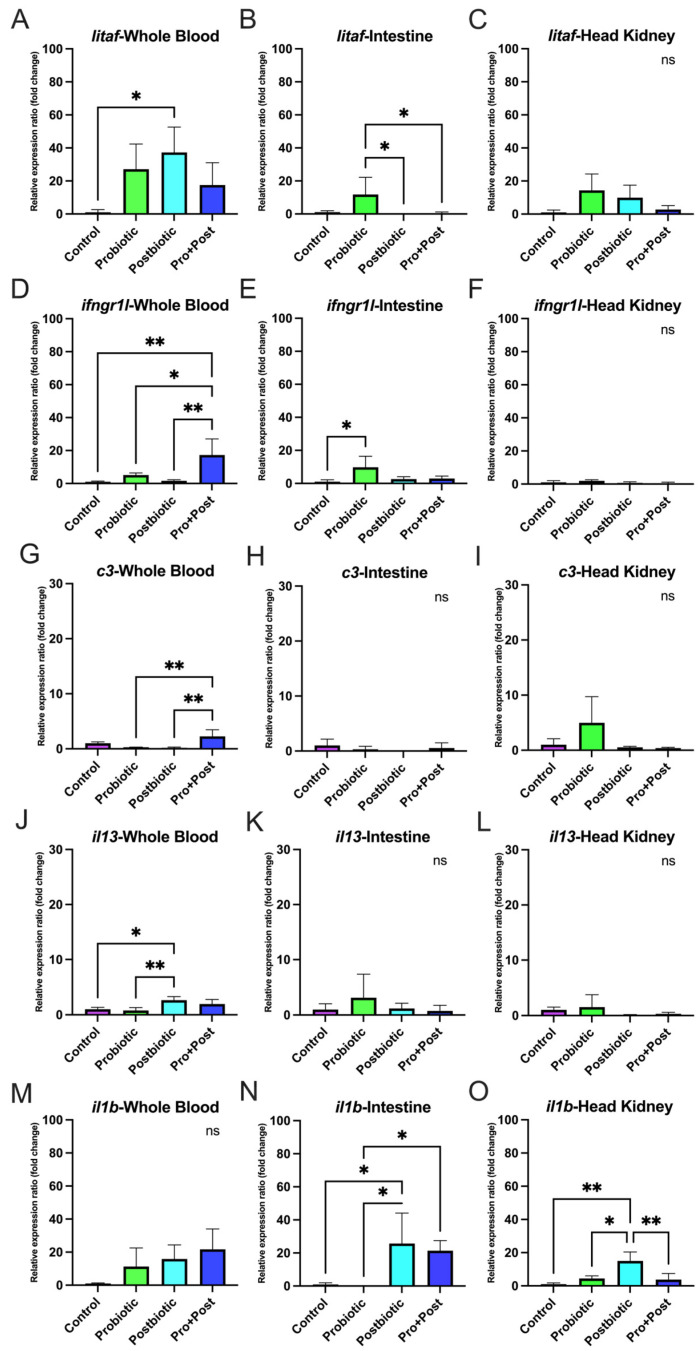
Relative proinflammatory-related gene expression (fold change) including *litaf* (**A**–**C**), *ifngr1l* (**D**–**F**), *c3* (**G**–**I**), *il13* (**J**–**L**), and *il1b* (**M**–**O**) in head kidney, intestine and whole blood of *P. hypophthalmus* following 30-day supplementation with probiotic, postbiotic, and their combination derived from *B. subtilis* AAHM-BS2360 after being challenged with *E. tarda*. Data are represented as mean ± SD (n = 8). Asterisks indicate statistically significant differences (ns = not significant). * (*p* < 0.05), ** (*p* < 0.01).

In whole blood, the Postbiotic group showed significantly higher *litaf* and *il13* expression than the Control group (*p* < 0.05), and the *il13* gene was also significantly elevated compared to the Probiotic group (*p* < 0.01). The Pro + Post group significantly upregulated *ifngr1l* (*p* < 0.01; *p* < 0.05) and *c3* (*p* < 0.01) compared to other treatments. No significant differences in *il1b* expression were observed across treatments in whole blood (*p* > 0.05) (Figure 9A,D,G,J,M).

In the intestine, the Probiotic group significantly increased *litaf* expression compared to the Postbiotic and Pro + Post groups (*p* < 0.05), and *ifngr1l* compared to the Control group (*p* < 0.05). The Postbiotic group significantly upregulated *il1b* compared to the Control and Probiotic groups, while the Pro + Post group also showed higher *il1b* expression than the Probiotic group (*p* < 0.05). No significant differences were found in *c3* and *il13* expression in the intestine (*p* > 0.05) (Figure 9B,E,H,K,N). The investigation revealed that the gene expression levels of *litaf*, *ifngr1l*, *c3*, and *il13* in all treatment groups were not significantly different (*p* > 0.05), which was a notable observation in the head kidney. However, a significant upregulation of the *il1b* gene was observed in the Postbiotic treatment group in comparison to all alternative treatments (*p* < 0.05; *p* < 0.01) (Figure 9C,F,I,L,O).

### 3.7. Growth-Related Gene Expression

**Figure 10:**
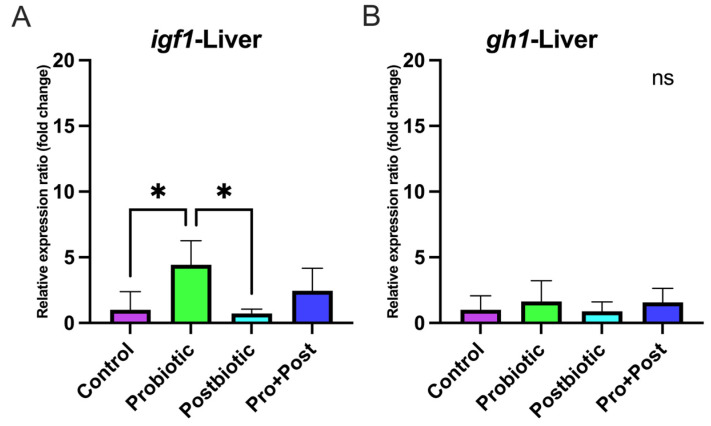
Relative growth-related gene expression (fold change) including *igf1* (**A**) and *gh1* (**B**) in the liver of *P. hypophthalmus* following 30-day supplementation with probiotic, postbiotic, and their combination derived from *B. subtilis* AAHM-BS2360. Data are represented as mean ± SD (n = 8). Asterisks indicate statistically significant differences (ns = not significant). * (*p* < 0.05).

The results of the qRT-PCR analysis revealed a significant increase in the activity of the *igf1* gene in the Probiotic treatment group in comparison to the Control and Postbiotic treatments (*p* < 0.05) (Figure 10A). However, the *gh1* gene expression did not show significant differences in all treatments (*p* > 0.05) (Figure 10B).

### 3.8. Growth Performance Analysis

The Probiotic and Pro + Post treatment groups significantly increased the SGR value compared with the Control group (*p* < 0.05). The Pro + Post group had a significantly higher ADG value than the Control group (*p* < 0.05). Despite the absence of a statistically significant difference in FCR values across all treatments (*p* > 0.05), the group’s treatment containing *B. subtilis* AAHM-BS2360 exhibited superior performance in terms of growth and feed efficiency, as evidenced by the average values. The data are presented in Table 2.

### 3.9. Histopathology

**Figure 11:**
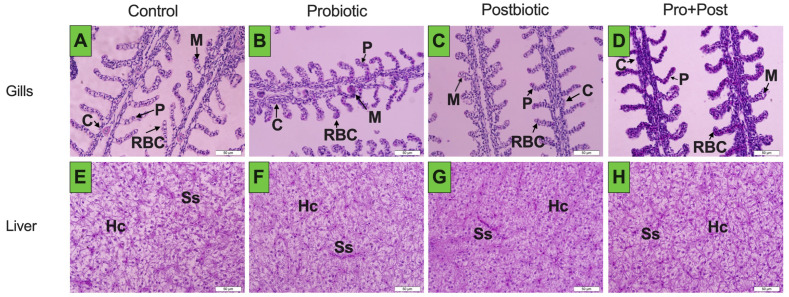

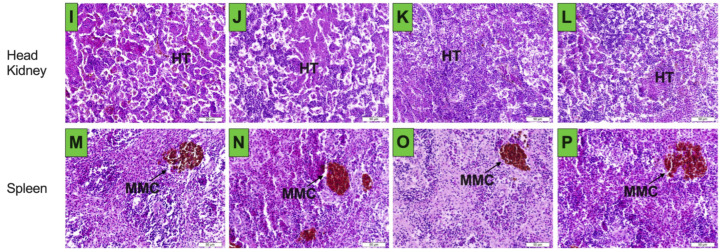

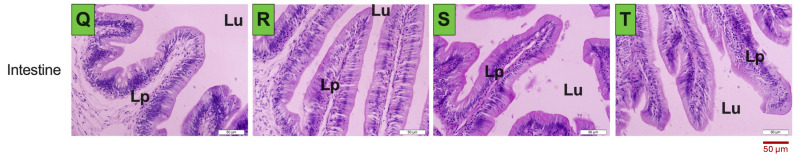
Histopathology of gills (**A**–**D**), liver (**E**–**H**), head kidney (**I**–**L**), spleen (**M**–**P**), and intestine (**Q**–**T**) of *P. hypophthalmus* following 30-day supplementation with probiotic, postbiotic, and their combination derived from *B. subtilis* AAHM-BS2360. Pillar cells (P), mucous cells (M), chloride cells (C), erythrocytes/red blood cells (RBC), hepatocyte (Hc), sinusoid (Ss), melano-macrophage center (MMCs), hematopoietic tissue (HT), lumen (LU), lamina propria (LP).

The histopathological examination of the gills demonstrated the absence of proliferation and hyperplasia of mucous cells, characterized by a normal lamellae structure. (Figure 11A–D). The liver analysis revealed unremarkable hepatocyte nuclei and an absence of congestion in the sinusoids, though low levels of hydropic degeneration were observed (Figure 11E–H). The head kidney exhibited normal conditions devoid of inflammation or necrosis of the hematopoietic tissue (Figure 11I–L). The melano-macrophage centers (MMC) in the spleen exhibited immune response activity in all treatments, devoid of any observed congestion or lymphoid depletion (Figure 11M–P). The intestinal villi were observed to be intact, characterized by a clear epithelial lining and goblet cells. This observation is indicative of optimal tissue health and structural integrity (Figure 11Q–T).

### 3.10. Survival Rate Analysis Following Post-Challenge Against E. tarda

**Figure 12:**
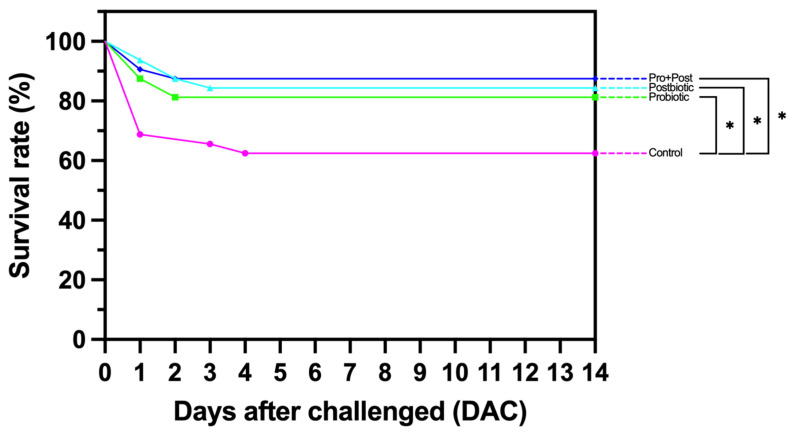
Survival rate (SR) (%) of *P. hypophthalmus* following 30-day supplementation with probiotic, postbiotic, and their combination derived from *B. subtilis* AAHM-BS2360 after intraperitoneal injection with *E. tarda*. Asterisks indicate statistically significant differences (*p* < 0.05).

The survival rate analysis revealed that the Pro + Post treatment group exhibited the highest survival rate of 87.5%, which was significantly different from the Control group with a value of 62.5% (*p* < 0.05). The Postbiotic and Probiotic groups demonstrated a survival rate of 84.37% and 81.25%, respectively, which was significantly different from the Control group (*p* < 0.05) (Figure 12).

## 4. Discussion

This study demonstrates the potential of *Bacillus subtilis* AAHM-BS2360 and its postbiotic metabolites as functional feed additives that improve growth performance, modulate immune responses, and reduce oxidative stress in *P. hypophthalmus* challenged with *Edwardsiella tarda*. No significant alterations in most serum biochemical parameters suggest that *B. subtilis* AAHM-BS2360 and its derivatives are safe and non-toxic. Serum biochemical profiles provide valuable information regarding the health status of pangasius and act as a monitoring tool for significant physiological stress or organ dysfunction in pangasius [33,34]. However, a noteworthy exception was observed for the AST/ALT ratio, which exhibited a considerable increase in the Postbiotic group, primarily attributable to the markedly augmented levels of AST as opposed to the comparatively lower levels of ALT.

In addition, a low range of ALT levels was also found in the 10 mg/kg ethanolic Kratom crude extract treatment in tilapia, the multivalent oral vaccine hydrogel in Asian seabass, and the treatment yeast extract supplemented feed in pangasius [29,35,36]. An increase in the AST/ALT ratio may reflect a transient hepatic response to bioactive compounds. Elevated AST/ALT ratios have been linked to an increase in oxidative stress conditions in the liver of fish and show the accumulation of microvacuoles, indicating lipid deposits in the liver [37,38]. Lipid peroxidase is closely associated with oxidative stress conditions. The extent of damage can be quantified by measuring malondialdehyde (MDA) levels and oxidative stress genes in the target organs [39]. The MDA enzyme analysis in the serum samples of the Postbiotic group showed a significant increase compared to the Control group. However, the MDA levels in the liver of the Control group were more dominant. To verify the physiological condition of the liver, histopathological analysis was also performed, which provided an overview of the health of the fish liver. Excessive lipid consumption can lead to increased lipid deposition in the liver, resulting in cell swelling, nuclear translocation, accumulation of lipid droplets, and an increase in hepatocyte diameter [40]. In contrast, the benefits of probiotics and their postbiotic metabolites in alleviating hepatic lipid accumulation and oxidative stress by augmenting uridine circulation are noteworthy [41]. Histological analysis of the liver revealed no evidence of lipid droplets or fatty degeneration. In general, the organs were in satisfactory condition and did not exhibit substantial tissue damage.

A significant increase in lysozyme activity in mucus samples from both the probiotic and postbiotic groups indicates enhanced mucosal immunity. Lysozyme is a key innate immune enzyme that hydrolyzes bacterial cell walls, offering first-line protection against pathogens [42]. These findings suggest that the postbiotic metabolites of *B. subtilis* AAHM-BS2360 may enhance the synthesis of antimicrobial peptides, lysozyme, or beneficial microbial metabolites within the mucus thereby impeding the proliferation of pathogens [16]. These mechanisms encompass stimulation of innate immunity factors, production of antimicrobial metabolites, modulation of skin microbiota, activation of mucosal immunity, and enhancement of barrier integrity [43,44,45]. A comparable enhancement of lysozyme activity in mucus has been observed in the context of probiotic *Pediococcus acidilactici* feeding in tilapia [46]. Although bactericidal activity did not differ significantly among groups, the upregulation of innate immune-related genes such as *lygl1* and *b2ml* suggests immunomodulatory effects at the molecular level [47,48].

The decline in MDA levels across the liver, intestine, gills, and skin samples in the treatment group, in conjunction with the incorporation of *B. subtilis* AAHM-BS2360 components in the feed, signifies a reduction in lipid peroxidation, a process initiated by reactive oxygen species (ROS), including superoxide radicals, hydrogen peroxide, and hydroxyl radicals. MDA, being reactive, can interact with proteins, DNA, and lipids, resulting in cellular damage and apoptosis. Increased MDA levels lead to reduced growth performance of pangasius, attributed to a decrease in antioxidant function and inflammatory response, which are biomarkers of oxidative stress in pangasius [49,50]. Similarly, Liu et al. [51] also showed an increase in MDA in striped catfish due to excessive histamine exposure. In addition to the decrease in MDA levels, the *B. subtilis* AAHM-BS2360 component in the target organ also increased catalase (CAT) activity. The Probiotic group showed increased CAT activity in the liver, the Pro + Post group showed increased CAT in serum, and the Postbiotic group showed increased CAT levels in the gill. Catalase (CAT) functions as an antioxidant enzyme that decomposes H_2_O_2_ into water (H_2_O) and oxygen (O_2_), thus preventing oxidative damage. Catalase cooperates with superoxide dismutase (SOD), which converts superoxide radicals (O_2_-) into H_2_O_2_, which is then neutralized by catalase. In conditions where catalase is present in low concentrations, glutathione peroxide (GPX) can also reduce H_2_O_2_ using glutathione (GSH). Catalase is essential in maintaining cellular redox balance, protecting cells from oxidative damage and maintaining physiological homeostasis [49,52]. In the present study, the treatment group of metabolite components of *B. subtilis* AAHM-BS2360 did not demonstrate a significant increase in the activity of other antioxidant enzymes, such as GPx, GR, GSH, GST, and SOD, when compared to the Control group. This may be attributed to the effective treatment in suppressing ROS production, thereby ensuring that other antioxidant enzymes do not need to be activated excessively, and the physiological regulation of the body adjusts the production of antioxidant enzymes as required, as shown in the study on the effects of probiotic, parabiotic, and postbiotic *B. subtilis* on Asian seabass and tilapia [53,54].

The finding of antioxidant enzyme activity is consistent with the results of the antioxidant gene expression analysis, which also exerted a positive effect on all treatment groups of *B. subtilis* AAHM-BS2360 metabolite components. In response to a subsequent challenge with *E. tarda*, both the Postbiotic and Pro + Post groups exhibited a predominant modulation of antioxidant gene expression in response to infection. The production of bioactive metabolites, such as peptides, exopolysaccharides, antioxidant enzymes, and surfactants, by *Bacillus* spp. has been demonstrated to enhance the activity of the internal antioxidant system in fish [55,56]. The regulation of key antioxidant genes and oxidative stress responses further supports the role of *B. subtilis* AAHM-BS2360 in providing effective protection in preventing oxidative stress in pangasius, which is consistent with previous reports of probiotic-mediated oxidative stress reduction in several fish species [54,57,58,59].

The present study investigates the modulatory effects of a dietary treatment containing *B. subtilis* AAHM-BS2360 on the expression of immune-related genes (*lygl1*, *tgfb*, *tnf*, *b2ml*) and proinflammatory genes (*litaf*, *ifngr1l*, *c3*, *il13*, *il1b*) in various target organs. This finding suggests that *B. subtilis* AAHM-BS2360 supplementation enhances both innate and adaptive immunity, exhibiting broad immunomodulatory effects, especially in enhancing non-specific immune responses, immune signaling and cytokine response, both post-feeding and challenge tests with pathogenic bacteria [60]. The upregulation of *lygl1*, *tgfb*, *tnf*, and *b2ml* gene expression in gills tissue in the Pro + Post group highlights the role of *B. subtilis* AAHM-BS2360 in modulating mucosal immunity, which is critical for protecting fish from waterborne pathogens [61]. In addition, the upregulation of immune genes was also seen to be dominant in the Postbiotic treatment after the challenge test with *E. tarda*. The Pro + Post group also demonstrated a marked tendency for proinflammatory gene expression activity. The probiotic strain *Pseudomonas aeruginosa* FARP72, administered at 10^8^ cells/g of feed in pangasius, significantly enhanced the innate immune response and proinflammatory gene. This was evident in increased lysozyme activity after 30 days and elevated gene expression of *c3* and *il1b* in liver and kidney organs after 1 day of intraperitoneal injection with *E. tarda* [62]. In summary, potential therapeutic interventions like dietary modification, probiotics, postbiotics, and gut microbiota transplantation may reduce oxidative stress and inflammation linked to metabolic syndrome by altering the gut microbiota and epigenetic changes [63,64].

The present study set out to assess the impact of a feeding formulation containing cell components and metabolites of *B. subtilis* AAHM-BS2360 on the growth of pangasius. Supplementation with *Bacillus subtilis* AAHM-BS2360 metabolite components significantly impacted specific growth rate (SGR), particularly in the Probiotic and Pro + Post groups. This effect was corroborated by the significant upregulation of the *igf1* gene expression in the Probiotic group. The expression of *igf1* gene provides valuable insights into the molecular mechanisms that stimulate tissue growth, offering a comprehensive understanding of successful aquaculture [65]. Furthermore, an increase in % SGR was demonstrated following 60 days of feeding probiotic *B. aerius* B81e to *Pangasius bocourti* [66]. *Bacillus subtilis*-supplemented feed was also reported to have a positive impact on the growth performance of hybrid grouper [67].

Following the challenge posed by the pathogenic bacteria *E. tarda*, the Pro + Post group demonstrated the highest survival rate, reaching an impressive 87.5%. This was closely followed by the Postbiotic group, which achieved a survival rate of 84.37%. The Probiotic group also performed exceptionally well, with a survival rate of 81.25%. To provide a point of comparison with the present study, a challenge test against *Aeromonas hydrophila* FW52 in *Pangasius bocourti* using probiotic *Bacillus aerius* B81e resulted in an SR of 77.3% [66], while probiotic *B. velezensis* JW achieved an SR of 80% against *A. hydrophila* in *Carassius auratus* [68]. Furthermore, a probiotic mixture comprising *Bacillus subtilis*, *Enterococcus faecium*, *Lactobacillus plantarum*, and *Saccharomyces cerevisiae* has been shown to yield an SR of 60% in pangasius fry in response to *A. hydrophila* injection [69]. In a similar study, the use of *Bacillus subtilis* CFS in Asian seabass led to an SR of 69.3% [53]. These comparative data demonstrate the superior protective effects of the combination of postbiotic metabolite and probiotic *B. subtilis* AAHM-BS2360 against pathogenic infection in pangasius.

## 5. Conclusions

Overall, these findings underscore the potential of *B. subtilis* AAHM-BS2360 as a functional feed additive in pangasius aquaculture. The supplementation of *B. subtilis* AAHM-BS2360 as probiotics resulted in a significant enhancement of growth performance and immune gene expression. In contrast, the supplementation of the postbiotic metabolite component of *B. subtilis* AAHM-BS2360 demonstrated a remarkable augmentation in lysozyme activity within the mucus, which serves as the fish’s innate immune barrier against pathogens. Furthermore, the post-challenge response with *E. tarda* revealed an upregulation of immune and proinflammatory genes. The combination of probiotics and postbiotics (Pro + Post) offered broad-spectrum immunomodulatory benefits, which could be advantageous in disease management strategies. It excelled in modulating proinflammatory and antioxidant gene expression, significantly dominating the challenge test with the highest SR. Future research should explore the long-term effects of *B. subtilis* AAHM-BS2360 supplementation and its potential application to other aquaculture species.

## Figures and Tables

**Table 1:** Primers used in qRT-PCR analysis of gene expression.

Gene Group	Genes	Nucleotide Sequences (5′ → 3′)	Tm (°C)	Efficiency (%)	Accession Number
Housekeeping gene	*Actin*, *beta 1* (*actb1*)	F-5′-GAGCGCAAGTACTCTGTATGGA-3′	60	98.5	XM_026928832.3
		R-5′-CTGTGGTGGTTACAGTCCTGTT-3′			
	*18S rRNA* (*rna18s*)	F-5′-TGACTCAACACGGGAAACCTC-3′	60	102.4	XR_004577708
		R-5′-CAGACAAATCGCTCCACCAAC-3′			
Stress oxidative and Antioxidant-related gene	*Copper-only SOD repeat protein* (*cusr*)	F-5′-GTCCATCTTACCCGGTGCCC-3′	60	99	XM_034299545.1
		R-5′- CGAGAGAAGACCCGGAACGC-3′			
	*Catalase* (*cat*)	F-5′-AGCAGGCGGAGAAGTACCCA-3′	60	104.7	XM_026919141.2
		R-5′-GCTGCTCCACCTCAGCGAAA-3′			
	*Glutathione peroxidase 3* (*gpx3*)	F-5′-GTCACTGCAGGATGCAACAC-3′	60	106.3	XM_026947312.2
		R-5′-TTGGAATTCCGCTCATTGAT-3′			
	*Heat shock protein 70 family*, *member 13* (*hspa13*)	F-5′-CTCCTCCTAAACCCCGAGTC-3′	60	100.4	XM_026934573.2
		R-5′-CCACCAGCACGTTAAACACA-3′			
	*Nitric oxide synthase 1* (*neuronal*) (*nos1*)	F-5′-ACACCACGGAGTGTGTTCGT-3′	60	99.6	XM_026931613.2
		R-5′-GGATGCATGGGACGTTGCTG-3′			
Immune-related gene	*Lysozyme g-like 1* (*lygl1*)	F-5′- TTTTTGGAGACGTCACAAAGATCG-3′	60	101.3	XM_026940542.3
		R-5′- TGGTGATGATGTTCTTGTACTGCT-3′			
	*Transforming growth factor*, *beta* (*tgfb*)	F-5′-GAACACTGTCCTCCTTGTCCTC-3′	60	93.4	XM_026909968.3
		R-5′-TCGTATTTGGTGGTGAGGATGG-3′			
	*Tumor necrosis factor* (*tnf*)	F-5′-AGTGCAAAGTCAAAAAGCGAGG-3′	60	100.2	XM_026921878.3
		R-5′-TGATACTTGGAGCCAGAGCATC-3′			
	*Beta-2-microglobulin*, *like* (*b2ml*)	F-5′-AGAGAACACACTGATCTGCCAC-3′	60	94.6	XM_026925487.3
		R-5′-GCGACGCTCTTAGTCAGATGAA-3′			
Proinflammatory-related gene	*Lipopolysaccharide-induced TNF factor* (*litaf*)	F-5′-TGCATTATTGGCTGTATGTATGGC-3′	60	95.1	XM_026930110.3
		R-5′-GTGGATATGTGCTCAGTTCCTGTT-3′			
	*Interferon gamma receptor 1-like* (*ifngr1l*)	F-5′-TACATAACAGTGGAAGCTCAACCA-3′	60	90.2	XM_026939395.3
		R-5′-AAGTTTGTGTTGTTGGACGGAAAT-3′			
	*Complement C3* (*c3*)	F-5′-GAATAGCTCCTGACTGTCCCAC-3′	60	98.3	XM_034300087.2
		R-5′-ATGTGTAGCCCAGTCTGTTCTG-3′			
	*Interleukin 13* (*il13*)	F-5′-GTTTATATCCGGCTTTCATGTGCA-3′	60	102.3	XM_034311451.2
		R-5′-AGAAAAACAACCACGACCTTCATC-3′			
	*Interleukin 1*, *beta* (*il1b*)	F-5′-CTATTCTGCTGGCCATTACTCTGA-3′	60	100.8	XM_034312378.2
		R-5′-ATGAGAGAAAGAGGTTGCTCTTCA-3′			
Growth-related gene	*Growth hormone 1* (*gh1*)	F-5′-CCCAGCAAGAACCTCGGCAA-3′	60	94.1	GQ859589.1
		R-5′-GCGGAGCCAGAGAGTCGTTC-3′			
	*Insulin-like growth factor 1* (*igf1*)	F-5′-GCAACGGCACACAGACACGC-3′	60	96.2	XM_034313382.2
		R-5′-CAGACGTTCCCTCACCATCCTCT-3′			

**Table 2:** Growth performance of *P. hypophthalmus* after 30 days of dietary supplementation with probiotic, postbiotic, and their combination derived from *B. subtilis* AAHM-BS2360.

Growth Parameter	Treatment			
	Control	Probiotic	Postbiotic	Pro + Post
Spesific growth rate (SGR); %/day	0.74 ± 0.43 ^a^	2.81 ± 0.61 ^b^	2.61 ± 0.79 ^ab^	3.29 ± 0.98 ^b^
Average daily gain (ADG); g/day	0.37 ± 0.23 ^a^	1.31 ± 0.35 ^ab^	1.24 ± 0.40 ^ab^	1.42 ± 0.52 ^b^
Feed conversion ratio (FCR)	1.06 ± 0.35 ^a^	0.79 ± 0.04 ^a^	0.77 ± 0.05 ^a^	0.81 ± 0.13 ^a^

Data (Mean ± SD) in same row with different letters are significantly different (*p* < 0.05).

## Data Availability

The data presented in this study are available in this manuscript.
